# Nanocomposite orthopaedic bone cement combining long-acting dual antimicrobial drugs

**DOI:** 10.1016/j.bioadv.2023.213538

**Published:** 2023-10

**Authors:** Lirong Yang, Abdulla A. Yergeshov, Yazan Al-Thaher, Svetlana Avdokushina, Evgeny Statsenko, Timur I. Abdullin, Polina Prokopovich

**Affiliations:** a School of Pharmacy and Pharmaceutical Sciences, Cardiff University, Cardiff, UK; b Institute of Fundamental Medicine and Biology, Kazan (Volga Region) Federal University, 18 Kremlyovskaya St., 420008 Kazan, Russia; c Institute of Geology and Petroleum Technologies, 4/5 Kremlyovskaya St., 420111 Kazan, Russia

**Keywords:** PJI, Gentamicin, Chlorhexidine, Bone cement, PBAE, Silica nanoparticles, Orthopaedics

## Abstract

Antibiotic loaded bone cements are widely used in total joint replacement (TJR); despite many limitations such as a burst release which leads to antibiotic concentration below inhibitory levels and possibly contributing to the selection of antibiotic resistant strains. In order to address such limitations and to simultaneously address antibiotic resistance and short-term antimicrobial activity, we developed a nanocomposite bone cement capable of providing a controlled release of antimicrobial agents from bone cement to act as prophylaxis or treatment against prosthetic joint infections (PJIs).

Gentamicin and chlorhexidine were loaded in combination on silica nanoparticles surface using layer-by-layer coating technique (LbL) combining hydrolysable and non-hydrolysable polymers.

The drug release from the nanocomposite continued for >50 days at concentrations higher than the commercial formulation containing the same amount of antimicrobial drugs, where burst release for few days were observed. Moreover, the nanocomposite bone cement showed superior antimicrobial inhibition without adversely affecting the mechanical properties or the ability of osteoblasts to grow. In vivo experiments with an infected bone lesion model along with mass-spectrometric analysis also provided further evidence of efficacy and safety of the implanted nanocomposite material as well as its prolonged drug eluting profile.

The developed nanocomposite bone cement has the potential to reduce PJIs and enable treatment of resistant established infections; moreover, the newly developed LbL based nano-delivery system may also have wider applications in reducing the threat posed by antimicrobial resistance.

## Highlights

• The nanocomposite cement has a dual antimicrobial effect;
• This cement material showed superior antimicrobial activity against different gentamicin resistant clinical strains.
• The nanocomposite is cytocompatible with non inferior mechanical properties compared to the commercial product.
• The developed cement is safe and effective as demonstrated in vivo using an animal model.

## Graphical abstract

**Figure:**
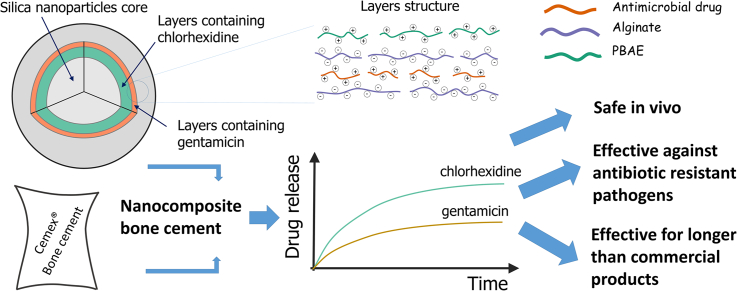

## 1 Introduction

The use of antibiotic laden bone cements (ALBCs) has become a well-established practice to prevent prosthetic joint infections (PJIs) after joint replacement surgery [1,2]. However, large amounts of antibiotics need to be added to achieve therapeutic levels (up to 1 g per 40 g of cement) [3]. There are several problems related to the release profile of antibiotics from ALBCs. The burst release in the first few hours after surgery, followed by low release below inhibitory levels which does not provide prophylaxis for a long period of time [[4], [5], [6]]. Also, the sub-inhibitory concentrations of the released drug add to the problem of emerging antibiotic resistant bacterial strains [7,8]. In addition, >90 % of the loaded antibiotics remain entrapped inside the cement matrix [8,9]. Peak mean concentrations of antibiotic released from PMMA beads and spacers is reached in the first hours after implant and fall below detection limits after 1–2 weeks [10]; however, PJI can occur months and years after surgery [11] demonstrating how the antimicrobial window of the current products is not satisfactory. As PJIs are both an economic burden for the health care providers and dramatically affect patients' lives [[12], [13], [14], [15]], the development of new bone cement products that exhibit a prolonged antimicrobial activity is of extreme urgency.

The limitation of antibiotic laden bone cements are also compounded by the emergence of resistant bacterial pathogenic strains that has become a significant global health threat [16,17]. The lack of innovative approaches for the treatment of antibiotic drug resistant bacteria is severely affecting many fields in medicine, including surgery, cancer chemotherapy, sepsis etc. [18]. Also, adding to the complexity of this problem is the lack of investment in antibiotic drug discovery by pharmaceutical companies because of low return rates compared to chronic diseases drug targets [19,20]. The development of new classes of antibiotic is a possible approach for this problem, however, few classes have been introduced over the last two decades. Furthermore, significant resistance can develop in a period of months to years after the introduction of new antibiotic for clinical use [21]. For example, after the introduction of daptomycin for clinical use in 2003, resistance in patients was observed with *Enterococcus faecium* and MRSA infections within less than year. Consequently, finding alternative approaches to controlling bacterial infections is urgently needed [22].

Many causative organisms in prosthetic infections have been reported to be resistant to certain antibiotics [23]; it was reported that 41 % and 66 % of *Staphylococci* isolates, taken from patients with prosthetic joint infections, were resistant to gentamicin and tobramycin, respectively [24,25]. Also, the resistance is significantly higher in patients with previous use of ALBC, which indicates the selection of aminoglycoside resistant strains after using ALBC [26,27]. Polymicrobial infections have been increasingly detected with complex microbiological treatment and poor clinical outcome [24].

One of the approaches for the treatment of antibiotic resistant strains is the use of drug combinations to effectively eradicate the multi-drug resistant phenotypes [28]. This approach includes antibiotic-antibiotic combinations to either directly target resistant mechanisms or to provide more than one mode of action by targeting different sites in bacterial cell [29]. Combination antibiotic therapy provides many advantages as compared to monotherapy such as a broader antibacterial spectrum, synergistic effects and minimizes the risk for emerging resistance during therapy [28]. Also, combinations are increasingly used to improve the efficacy of available drugs against multidrug resistant strains [22]. However, the use of combination antimicrobial therapy is associated with increased risk of side effects i.e. ototoxicity and nephrotoxicity, especially when taken systemically [30]. Therefore, it is recommended to use the most selective single agent as soon as the antibiotic susceptibility profile of the causative agent is known, or using a local delivery whenever possible to reduce drugs concentration in the systemic circulation and their consequent side effects [31].

In this study, we tackled simultaneously two of the current limitations associated to ALBC, the short duration of the antimicrobial activity and the inactivity against antibiotic resistant organisms. We hypothesized that layer-by-layer coatings using poly-beta-amino-esters coatings chlorhexidine and gentamicin containing could be developed on silica nanoparticles (NPs) and that this NPs, once incorporated in PMMA bone cements, could create a novel nanocomposite combination antimicrobial bone cement releasing both antimicrobial agents for prolonged periods of time. We have characterized the NPs and the material properties of the nanocomposite bone cement to ascertain functionality and then tested the material in vivo to validate both efficacy and safety.

## 2 Materials and methods

### 2.1 Chemicals

Triton X-100, Tetraethyl orthosilicate (TEOS), (3-Aminopropyl) triethoxysilane (APTS), sodium alginate, chlorhexidine diacetate, sodium acetate trihydrate, piperazine, gentamicin sulphate, phosphate buffer solution (PBS) tablets, o-phthaldialdehyde reagent were purchased from Sigma-Aldrich, UK.

Cyclohexane, 1-hexanol, ammonium hydroxide 35 %, acetonitrile, ethanol, methanol, glacial acetic acid and 1-propanol were purchased from Fishers, UK. All reagents were stored according to manufacturer's guidelines and used as received.

### 2.2 Poly-β-amino-esters synthesis

Amino-terminated poly(β-amino ester)s (PBAEs) were synthesized by mixing 3.7 mmol of Bisphenol A ethoxylate diacrylate and 4.1 mmol of piperazine in 5 ml of DCM. The polymerization was performed under stirring at 50 °C for 48 h. PBAEs were precipitated through pouring the reaction mixture in about 10 times the volume of diethyl-ether under vigorous mixing, after which the solvents were removed under vacuum [5,6,[32], [33], [34]].

### 2.3 Nanoparticle preparation

#### 2.3.1 Amino functionalised silica nanoparticles synthesis

Silica nanoparticles functionalised with amine groups (SiO_2_-NH_2_) were prepared in one-pot synthesis by hydrolysis of TEOS in reverse micro-emulsion and subsequent functionalization with amino group [35].

Typically, Triton X-100 (17.7 g) was mixed with 16 ml of n-hexanol, 75 ml of cyclohexane, and 4.8 ml of deionized water; 600 μl of NH_4_OH (29.6 %) were added when the solution became transparent. After sealing and stirring for 20 min, 1 ml of TEOS was added with stirring for further 24 h. The surface of the silica nanoparticles was functionalized with amino groups by adding 50 μl of APTS to the micro-emulsion under stirring and incubating for further 24 h. The silica nanoparticles were recovered adding 200 ml of ethanol and centrifuging at 14000 rpm for 10 min (LE-80 K, Ultracentrifuge, Beckman Coulter, UK) at 20 °C (35,280 g). The nanoparticles were washed three times with deionized water and then left to dry at room temperature in a fume hood for 24 h [5,6,32].

#### 2.3.2 Layer by Layer (LbL) coating technique

Ten quadruple layers of a repeating sequence of (sodium alginate/chlorhexidine or gentamicin/sodium alginate/PBAE) were deposited on the silica NPs using the LbL technique. Polyelectrolytes and drugs were dissolved in acetic acid‑sodium acetate buffer (pH = 5 and 100 mM) in concentration of 2 mg/ml and 10 mg/ml respectively. Silica nanoparticles (~1 g) were placed in a test tube with 20 ml of sodium alginate solution; after stirring for 10 min, the dispersed NPs were centrifuged (5 min at 1300 *g*). The supernatant was disposed and replaced with 20 ml of fresh acetic acid‑sodium acetate buffer. Then, the NPs were resuspended and buffer was centrifuged and removed. 10 ml of chlorhexidine or gentamicin were stirred with the NPs for 10 min, centrifuged, and washed again with fresh buffer. Sodium alginate solution was then layered and washed. Finally, 20 ml of the PBAE solution were used. This sequence was repeated for each individual quadruple layer [6].

The following chlorhexidine and gentamicin layer combination were prepared):

• 9:1, where the first 9 inner quadruple layers contained chlorhexidine and the 10th outermost layer contained gentamicin.
• 8:2, where the first 8 inner quadruple layers contained chlorhexidine and the 9th and 10th layers contained gentamicin.
• 7:3, where the first 7 quadruple inner layers contained chlorhexidine and 8th, 9th and 10th outermost layers contained gentamicin.

### 2.4 Nanoparticle characterisation

#### 2.4.1 TEM

The shape and size of the prepared nanoparticles were determined through transmission electron microscopy (TEM). A droplet (4 μl) of nanoparticles suspension in water was placed on a plain carbon-coated copper TEM grid and left to evaporate in air under ambient laboratory conditions for few hours. Bright field TEM images at a magnification of 100,000 X were taken using a JEOL-1010 microscope at 80 kV equipped with a Gatan digital camera. Images were analyzed with ImageJ software to measure the diameters of at least 150 particles. [5,6]

#### 2.4.2 Thermogravimetric analysis (TGA)

Thermogravimetric analysis (TGA) was performed for different types of nanoparticles to study the build-up of LbL coatings and determine the organic content on the surface of the nanoparticles. TGA was performed using a Perkin-Elmer TGA 4000 instrument. Samples were heated from 50 to 800 °C with a constant heating rate of 10 °C per minute. Sample weight was recorded and weight loss percentage of each sample was calculated relative to initial weight of sample, prior to heating. The organic and inorganic material percentages were calculated by subtracting the point at initial weight loss (%) up to when the line plateaus [5,6,32,34].

### 2.5 Bone cement preparation

Bone cement preparation was carried out according to manufacturer's instructions and the ISO5833:2002 [36]. Both components of Cemex® (Tecres® S.p.A., Italy) bone cement were stored at recommended conditions (20–25 °C for the powder and 8–15 °C for the liquid in the dark) and conditioned to room temperature (23 °C) 2 h before mixing. NPs were added to the powder component and sifted before weighing and mixed thoroughly, while each liquid component was weighed separately. Finally, both components were hand-mixed in a polypropylene bowl with a polypropylene spatula for 1 min, before being poured into a polytetrafluoroethylene (PTFE) mould. Two types of moulds were used to produce samples with specific dimension for different tests. After applying the cement into the mould, the mould was clamped with two steel endplates covered with PTFE film at both ends. After 2 h, the samples were pushed out of the mould using a steel rod and allowed to cure for 24 ± 2 h at 23 °C. Samples were then sanded down to the correct dimensions using 320 grit silicon carbide paper.

### 2.6 Bone cement characterisation

#### 2.6.1 Rheology testing

The effect of adding the nanoparticles on the cement settling time was evaluated through rheological tests using Anton Paar MRC702 (Anton Paar Ltd., UK), equipped with 6 mm diameter circular flat plates. Dynamic oscillation tests were performed in these measurements, a sinusoidal oscillation strain (σ), of small amplitude (σ_0_) and frequency (ω), was applied to the sample:

$$\sigma \;\left(t\right)=\sigma_{0}\exp\left(\mathrm{i\omega t}\right)$$

The resulting stress (ω) was compared with the strain giving the complex modulus G*.

$$G^{*}=\frac{\sigma \;\left(t\right)}{\gamma \;\left(t\right)}$$

Because the two sinusoidal waves will have a phase difference, δ, the storage (*G*′) and loss modulus (*G*″) can be defined as the component in phase and π/2 out of phase with the strain, respectively.

$$G^{*}=G^{\prime }+iG^{\prime \prime }$$

and

$$G^{\prime }=\left|G^{*}\right|\;\cos\delta$$

$$G^{\prime \prime }=\left|G^{*}\right|\;\sec\delta$$

Rheological testing was conducted using dynamic time sweep at room temperature, plate distance 1 mm, a strain of 0.1 % and fixed frequency of 1 rad/s. For all tests, the bone cement solid phase was mixed with the liquid phase quickly with a spatula; the mixture was deposited onto the lower plate and experiments started as fast as possible. To account for the time elapsed during mixing and pouring, a timer was started at the start of mixing the liquid with powders. Measurement of complex Young's modulus and phase angle were taken every 6 s for up to 10 min. The setting time was extracted from each curve as the time correspondent to a local maximum of tan delta (G"/G'). Each sweep experiment was carried out on three independently prepared cement samples, and results are presented as mean and standard deviation [5,6,37].

#### 2.6.2 Drug release quantification

The different types of nanoparticles (7:3, 8:2 and 9:1) were added to Cemex® bone cement to achieve a concentration of chlorhexidine + gentamicin of 3 % *w*/w.

A PTFE mould was used to produce cylindrical samples with 6 mm diameter and 10 mm length. Each sample weighed 0.40 ± 0.01 g and three samples were used for release study from each type of bone cement. The bone cement samples were incubated in 3 ml sterile PBS buffer (pH 7.3) at 37 °C. The release media was replaced each day in order attain sink condition [5,6,32,34,37].

Gentamicin released was quantified through fluorescence spectroscopy using o-phthaldialdehyde reagent that reacts with the amino groups of the antibiotic producing a fluorogenic product. 70 μl of buffer were mixed with 70 μl of isopropanol and 70 μl of OPA reagent in a black 96-wells plate. After 30 min at room temperature in the dark, the fluorescence was determined (Ex = 340 nm and Em = 450 nm) with a fluoroscan (FLUOROstar Optina, BMG Labtech). Six independent standard solutions of gentamicin (concentrations ranging from 0 to100 μg/ml) were also prepared for the calibration curve and analyzed concurrently for each 96-well plate run [38].

The amount of chlorhexidine was determined using reverse-phase High Performance Liquid Chromatography with an Agilent Technologies® HPLC (1100 series) equipped with a Waters-Spherisorb ODS2 column (Pore size- 80 Å 5 μm and packing dimension of 4.6 mm × 150 mm) and a UV detector at 239 nm. The injection volume was 10 μl while the mobile phase was 0.1 M acetate buffer pH = 5 acetonitrile 58:42 at a flow rate of 1 ml/min. Stock solutions of chlorhexidine (ranging from 0.4 to 25 μg/ml) were prepared separately for calibration [34,37].

#### 2.6.3 Antimicrobial testing

Different stains from repositories and clinical isolates were tested. Gram-positive bacteria methicillin-resistant *Staphylococcus aureus* (NCTC12493), *Streptococcus pyogenes* (ATCC19615) and *Staphylococcus epidermidis* (ATCC 12228) along with Gram-negative bacterium *Acinetobacter baumannii* (NCIMB9214), *Pseudomonas aeruginosa* (NCIMB10548) and *Escherichia coli* (NCTC10418). The clinical isolates from anonymous PJIs patients in the period 2013–2015 (12 strains) were obtained from Bristol hospital and species were confirmed by polymerase chain reaction. Gentamicin resistance was defined as MIC >250 μg/ml.

Viable stocks were generated by spreading the frozen stock (stored at −80 °C in cryo-protective solution) on brain heart infusion (BHI) agar and incubating for 24 h at 37 °C. Cell cultures were prepared by inoculating a loopful of cells from an individual colony on the plates into sterile BHI broth followed by static incubation for 24 h at 37 °C. The cell suspension was diluted 1:1000 in fresh sterile BHI broth, and 20 μl of the diluted broth were added into a sterile 96-well plate. After that, each well was filled with 100 μl media of one of the bone cement samples for each day of release testing; the plate was then incubated for 18–24 h at 37 °C. The growth in each well was evaluated visually. Each data point was performed in triplicate for each individual strain on 6 individual batches of cements specimens, to determine the duration of the release media from the bone cement inhibitory activity towards bacteria growth as the day corresponding to the last daily release inhibiting bacterial growth [5,6,34,37].

#### 2.6.4 Mechanical testing

Compression tests were conducted using cylindrical specimens (diameter = 6 mm and height = 12 mm) and four-point bending tests were conducted using rectangular specimens (length, width, and thickness = 75 mm, 10 mm, and 3.3 mm, respectively). Fracture toughness tests were conducted using rectangular specimens (length, width, and thickness = 45.0 ± 0.1 mm, 10.0 ± 0.1 mm, and 3.3 ± 0.1 mm, respectively) with a sharp chevron notch (5.5 ± 0.5 deep) cut into the center of one of the long sides of the specimen using a sharp razor blade. The specimen was loaded at the center of the unnotched long face, in three-point bend mode (distance between the support rollers = 40 mm). All tests were conducted in accordance with ISO5833 [36] and ISO13586 [39] using a Zwick Roell ProLine table-top Z050/Z100 testing machine equipped with a dedicated TestXpert II software (Zwick Testing Machines, Herefordshire, UK); a crosshead speed of 20 mm/min was used for compression tests while a crosshead speed of 5 mm/min was used for bending and fracture toughness tests [5].

Compressive strength testing was performed after 24 h of bone cement preparation and after incubation in PBS for 3 months. For each combination of cement and mechanical test, 5 specimens were tested; when the bone cements were mixed with nanoparticles these were independent batches.

#### 2.6.5 Water uptake testing

Bone cement cylindrical samples were incubated in 3 ml PBS at 37 °C for 3 months; for the first 2 weeks, the samples were weighed daily; after that the samples were weighed every 3 days [5,6].

#### 2.6.6 Cytocompatibility of bone cement containing silica nanoparticles

##### 2.6.6.1 Cells

Saos-2 human osteosarcoma osteoblast-like cells (ATCC® HTB-85™) were cultured in RPMI-1640 medium supplemented with foetal bovine serum (10 % *v*/v) and 1 % v/v of a solution of penicillin (5000 U/ml)/streptomycin (5000 mg/ml). Cells were incubated at 37 °C in humidified atmosphere with 5 % CO_2_. Cells were grown till ~70 % confluence, washed twice with sterile PBS, and detached with trypsin; osteoblast cells were counted (using Trypan Blue to differentiate between viable and nonviable cells).

Bone cement samples (round disk with diameter = 10 mm and height = 5 mm) were placed in a 24-well plate, each bone cement sample was incubated in 1 ml of growth media inoculated with approximately 60,000 cells/well at 37 °C in humidified atmosphere with 5 % CO_2_ for up to 21 days; medium was replaced twice a week. Samples prepared without NPs were used as control.

##### 2.6.6.2 MTT

After a set period of incubation, the medium present in the well was removed and replaced with 1 ml of phenol red-free fresh medium. Twenty microliters of MTT reagent (5 mg/ml in PBS) were added to each well and the plate was incubated for 24 h at 37 °C in humidified atmosphere with 5 % CO_2_. 900 ml of the medium were removed from each well, 150 μl of dimethyl sulfoxide (DMSO) added to each well and the plate were incubated for further 10 min. Two hundred microliters of solution containing the dissolved formazan was transferred in a 96-well plate and analyzed with a spectrophotometer (Tecan® Infinite F50, Austria) at 560 nm.

##### 2.6.6.3 LDH

After a set period of incubation, the released LDH was determined transferring 75 μl of the cell culture media to 96-well plate for each sample with 50 μl of LDH enzymatic assay kit (Sigma-Aldrich, UK). The plates were covered with aluminium foil to protect from light and incubated at room temperature for 30 min. Then, the optical density was measured at a wavelength of 490 nm.

The total LHD was determined adding 75 μl of lysis solution to each sample in the 24-well plate containing cells with cement samples and incubated at 37 °C for 30 min. 75 μl cell culture media transferred to 96-well plate for each sample and analyzed as in for the released LDH.

##### 2.6.6.4 Calcium production assay-alizarin red

After 21 days of incubation of SaoS-2 cells on the bone cement samples, the medium was removed and replaced with 1 ml of glutaraldehyde 10 % (*v*/v) (Sigma-Aldrich, UK) and the plates were incubated for 15 min. Samples were then washed with deionized water three times. 1 ml of Alizarin Red S (10 g/l) (Sigma-Aldrich, UK) was added to each well and the plates were incubated for 20 min. After washing with deionized water, 1 ml of acetic acid 10 % (v/v) was added to each well and the plates were incubated for further 30 min. After this, 200 μl of solution was transferred in 96-well plate analyzed with a spectrophotometer (Tecan® Infinite F50, Austria) at 450 nm [5,37].

##### 2.6.6.5 NO

20 μl of Griess reagent (Molecular Probes, USA), 150 μl of fresh medium or nitrite standard and 130 μl of deionized water were mixed in a 96 well-plate and incubated for 30 min in the dark at room temperature. A photometric reference sample was prepared by mixing 20 μl of Griess regent and 280 μl of deionized water.

The absorbance of the samples relative to the reference sample was determined in a spectrophotometric microplate reader at wavelength 560 nm. Nitrite concentrations were determined using the calibration curve (nitrite solutions with concentrations between 1 and 100 μM) prepared at the time of each experiment.

### 2.7 In vivo study

Wistar rats were purchased from Vivarium of Academy of Medical and Technical Sciences (Russia). Animal care was performed according to the European regulations on the protection of experimental animals (Directive 2010/63/UE). The animals were maintained under recommended temperature and humidity with free access to water and full-ration certified feed. The selected rats (350 ± 35 g) were divided into experimental groups of 6 animals. Bone cement formulations were applied along with blank Cemex material to different thighs of the same animal in order to achieve more relevant assessment of the antibacterial drugs. The study protocol was reviewed and approved by the Institutional Animal Care Committee of the Kazan Federal University (protocol no.38 from 4.10.2022).

#### 2.7.1 Infected bone lesion

**Fig. 1:**
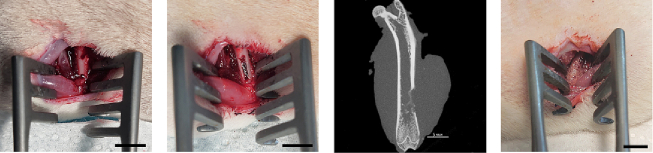
Representation of in vivo model. From left to right: surgical access to lateral surface of the femur bone and perforation of cavities; formation of longitudinal segmental defect in the compact bone and its inoculation with bacteria; X-ray visualization of the bone with the infected defect; treatment of the infected defect with bone cement material. Scale bar – 5 mm.

A modified femoral bone lesion in rats was established based on previous studies [40,41]. This model relates to post-traumatic osteomyelitis which requires appropriate therapy to be healed. Main steps of its implementation are illustrated in Fig. 1.

The rats were anesthetized by intraperitoneal injection of tiletamine–zolozepam–xylazine composition at a dose of 30–20–10 mg/kg, respectively. To alleviate pain, meloxicam solution (10 mg/ml 400 μl) was subcutaneously injected to each animal both during surgical interventions and afterward every other day. The primary surgical procedure included standard depilation and cleaning of the thigh surface at the lateral side, and incision of the skin. Furthermore, the connective tissues around biceps femoris and vastus lateralis muscles were dissected to provide access to lateral surface of the femur bone. Infected lesion was introduced within diaphysis area adjacent to the knee joint as follows (Fig. 1). Briefly, two cavities 2 mm in diameter with 5 mm distance between them were perforated using titanium bore under flushing with sterile isotonic solution. The area between cavities was mechanically dissected to create ca. 2 × 5 mm longitudinal excision in the compact bone. After complete stopping of bleeding with sterile gauze dressings, the bone defect was infected by *S. aureus* (ATCC29213). For this purpose, a night culture of *S. aureus* grown in Mueller-Hinton (MH) broth was diluted with sterile PBS to obtain a bacterial suspension (10^5^ CFU/ml). A 10 μl aliquot of the resulting inoculum was dropped into the bone lesion so that the inoculum was absorbed by underlying tissues. The dissected covering tissues were then sutured to temporary close the wounds. After maintaining the animals for 24 h to allow the infection propagation in the bone defect, the wounds were re-opened, and the bone cements were evenly applied by filling the lesion with the sample (65 mg). The dissected tissues were finally re-sewed using resorbable suture threads. The closed surgical wounds were cleansed by hydrogen peroxide solution (3 %) and monitored to ensure integrity of sutures and lack of acute purulent process.

#### 2.7.2 Swab test

The swab test was performed at days 2, 7, 14, 28 post-treatment. After providing a surgical access to each bone defect under anesthesia, sterile cotton swabs were moistened with 50 μl of sterile isotonic solution and gently applied to the wounds to absorb wound secretion. The inoculated swabs were then transferred into Stuart's transport medium and then into MH broth to release the bacteria. Colony-forming units (CFU/ml) in the resulting solution were quantified by spreading 5 μl of the inoculum at different dilutions on Petri dishes with MH agar, allowing them to grow for 24–48 h at 37 °C and counting individual colonies.

#### 2.7.3 X-ray microtomography (micro-CT)

The animals were sacrificed at days 14 and 28 post-treatment, and the thighs were harvested and fixed in 10 % neutral buffered formalin solution in PBS at room temperature for 72 h. The compact bone in diaphysis area around the lesion was evaluated by micro-CT technique using a Phoenix V|tome|X S 240 system. The shooting parameters were as follows: 100 kV, 110 μA, 200 ms detector integration time, mean thickness of each slice 0.4 mm, resolution 525 × 554 voxels with a voxel resolution 23 μm. For primary data processing and construction of a volumetric (voxel) model of the analyzed samples based on X-ray images (projections) the datos|x reconstruction software was conducted. The reconstructed 3D models were mathematically analyzed using an ImageJ 1.53q (Fiji) software with a BoneJ plugin. The following parameters of the entire bone in ROI were calculated: bone volume (BV), bone volume fraction (BV/TV), degree of anisotropy (DA), and fractal dimension (FD). In addition, bone mineral density (BMD) within the lesion area only using Hounsfield units [42] based on linear attenuation coefficients of air-fat-muscle [43], which were earlier adopted for CT-scanning. Avizo 8 software was used to obtain 3D images of reconstructed bones.

#### 2.7.4 Histological analysis

The pre-fixed femur bone explants were decalcified by 2 repetitive treatments with 10 % formic acid solution for 2.5 h each. The treated explants were washed in distilled water and dehydrated in a graded series of ethanol solutions (50, 70, 96, 100 %) and finally cleared in xylene. The samples were embedded in paraffin blocks and cut on a microtome HM 355S (Thermo Fisher Scientific) into 15 μm sections. The tissue sections were subjected to Giemsa, picro-Mallory trichrome and Von Kossa staining and analyzed using bright-field microscopy on an Axio Observer Z1 microscope (Carl Zeiss). Bacterial colonies in the sections were detected via Giesma staining and their relative amount was quantified using ImageJ software [34].

#### 2.7.5 LC–MS/MS analysis

To quantify chlorhexidine in blood plasma, liquid chromatography–tandem mass spectrometry (LC–MS/MS) analysis in multiple reaction monitoring (MRM) mode was performed using an Infinity 1290 HPLC system (Agilent) combined with a QTRAP 6500 triple quadrupole mass spectrometer (AB Sciex) with an electrospray ionization source (ESI).

Blood plasma samples were prepared from tail vein blood of the treated animals in dynamics and stored at −80 °C. The thawed samples were mixed with 3 volumes of methanol, briefly vortexed, incubated for 5 min and then centrifuged for 5 min at 16000 ×*g*. The deproteinated supernatants were collected, diluted with ultrapure water (1: 1), transferred into autosampler vials and subjected to LC–MS/MS analysis. The analytes were separated on a Discovery HS C18 column (3 μm, 5 cm × 2.1 mm, Supelco) using a gradient solution composed of 0.3 % formic acid in water (solvent A) and acetonitrile (solvent B). The gradient program was as follows: 0–2 min – 95 % of solvent A; 2–6.5 min – gradient 95–10 % of solvent A; 6.5–7.5 min – 10 % of solvent A; 7.5–7.6 min – gradient 10–95 % of solvent A; 7.6–10 min – 95 % of solvent A. The flow rate was 0.4 ml/min.

Electrospray ionization in positive ion mode was applied. The source parameters were as follows: capillary voltage 5.2 kV (−4.5 kV for negative ionization), gas 1 pressure 60 psi, gas 2 pressure 60 psi, curtain gas pressure 35 psi, temperature 500 °C. The quantifier/qualifier ions *m*/*z*, declustering potential, and collision energy were optimized for each analyte using an automated ‘Compound optimization’ algorithm of Analyst 1.6.2 software (AB Sciex). The selected quantitative / qualitative MRM transitions for chlorhexidine were 253.3 / 170.1 m/z (doubly-charged ion) and 505.1 / 353.1 m/z (singly-charged ion), respectively.

### 2.8 Statistical analysis

All results are expressed as means ± standard deviation (SD) from at least three independent values. The statistical significance between compared groups was by verified by one-way analysis of variance (ANOVA) followed by Tukey multiple comparison post-test (*p < 0.05, **p < 0.01, ***p < 0.001). Experimental results were considered statistically significant at 95 % confidence level (*p* < 0.05).

Multivariate analysis of variance (MANOVA) was carried out, using the R software [44] and lme4 package [45], to test the antimicrobial activity of the bone cement containing nanocarriers against the commercial formulation, where the multiple dependent variables were the number of days the growth was prevented, using the Pillai test. A paired-wise ANOVA comparison was further performed to determine the impact on individual species [6].

## 3 Results

### 3.1 Nanoparticles characterisation

**Fig. 2:**
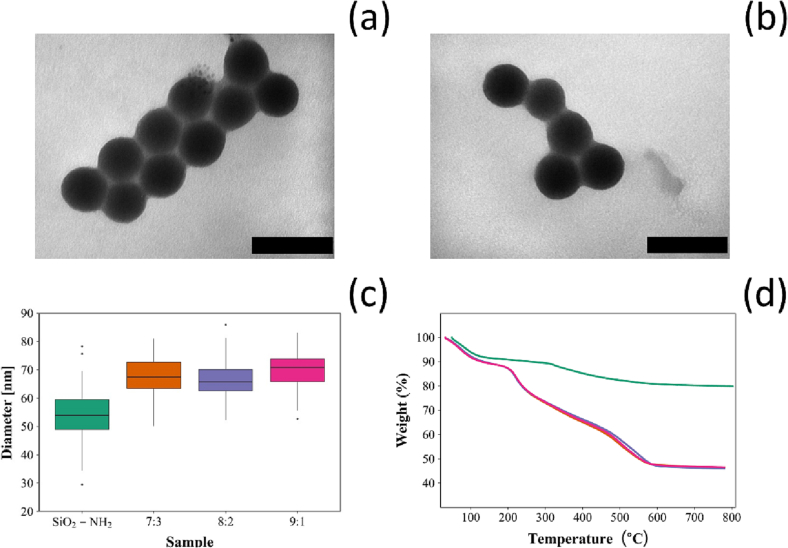
Examples of TEM images (Bar represent 100 nm) for coated silica nanoparticles 7:3 (a) and 9:1 (b); distribution of particles diameters (c) and TGA thermograms (d) ( uncoated SiO_2_-NH_2_,  7:3,  8:2 and  9:1).

Uncoated silica nanoparticles were round shaped (Fig. 2a and b) with a mean diameter of 54 ± 7 nm, after the deposition of 10 quadruple layers, the mean diameter increased to 68 ± 7 nm, 66 ± 6 nm and 69 ± 7 nm for 7:3, 8:2 and 9:1 NPs respectively (Fig. 2c).

TGA thermograms of uncoated silica NPs and of NPs with different number of quadruple layers are shown in Fig. 2d. For all, an initial weight loss around (5 %) was observed at about 100 °C; the organic content in each sample was calculated based on the weight loss beyond 100 °C, which truly corresponds to the combustion of organic matter [46].

The organic content for the amino functionalised silica NPs was 14.9 % (*w*/w). After the addition of 7 quadruple layers containing chlorhexidine, the organic content increased to 46.2 % (w/w). Depositing 10 quadruple layers on the surface of amino-functionalised silica NPs yielded similar organic content of around 58 % irrespectively of the number of layers containing chlorhexidine or gentamicin or chlorhexidine alone (*p* > 0.05).

### 3.2 Bone cement

#### 3.2.1 Drug release profiles

**Fig. 3:**
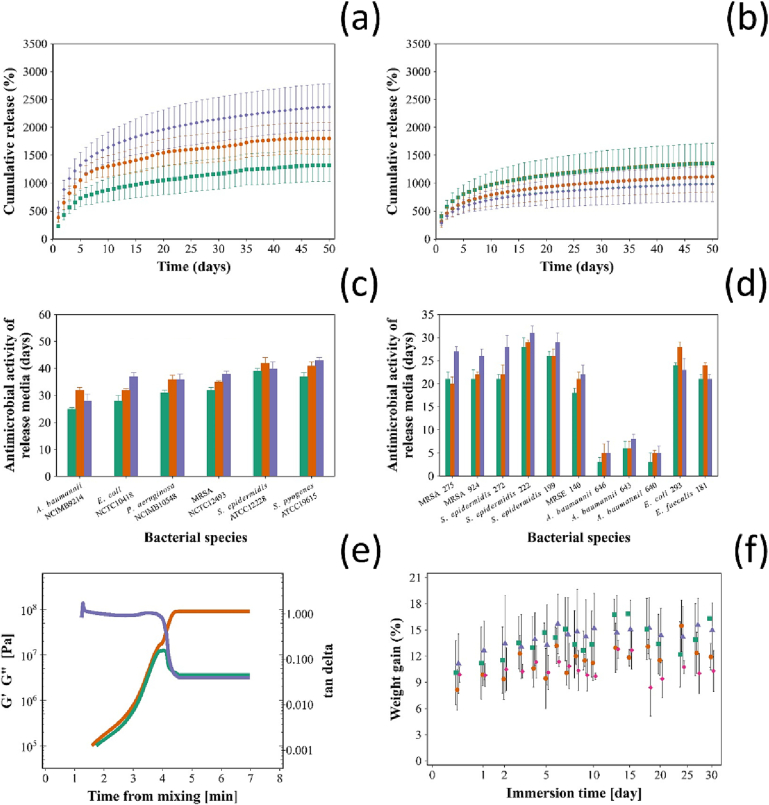
Chlorhexidine (a) and gentamicin (b) cumulative release from Cemex bone cement containing different types of NPs. Antimicrobial testing of Cemex cement containing different types of NPs against (c) catalogue strain and (d) clinical isolates ( 7:3,  8:2,  9:1) mean ± SD, *n* = 3). (e) Example of storage (G') () and loss (G") () modulus, tan δ () during bone cement settling for Cemex bone cement containing coated 9:1 NP. (f) Water uptake of Cemex () and Cemex containing different types of NPs ( 7:3,  8:2,  9:1) after incubation in PBS buffer, pH 7.3 (mean ± SD, n = 3).

All bone cements containing different types of NPs (7:3, 8:2 and 9:1) stopped releasing chlorhexidine and gentamicin after 50 days (Fig. 3a and b). Regardless of the antimicrobial agent and the type of NPs considered, the amount of drug released had a maximum at the initial contact with the aqueous solution and gradually decreased. There was a proportional increase in the cumulative release for gentamicin with increasing the number of layers containing gentamicin. Similarly, chlorhexidine release was also proportional to the number of chlorhexidine layers.

#### 3.2.2 Antimicrobial activity

The duration of the inhibition of the catalogue strains tested was significantly different among the different types of nanocomposites (*p* < 0.05); the release buffers from bone cements samples with embedded NPs containing only one quadruple layer with chlorhexidine exhibited antimicrobial activity when derived from greater number of days from the initial immersion in PBS (Fig. 3c). The antimicrobial activity was longest (up to 42 days) against *S. pyogenes* and *S. epidermidis.* The least antimicrobial activity was observed against *A. baumannii* for nearly 30 days. Clinical strains resistant to gentamicin inhibition was longer for samples with embedded NPs (*p* < 0.05) (Fig. 3d). The least inhibition duration was observed against the three *A. baumannii* clinical isolates (<7 days). The antimicrobial activity against other *Staphylococci* and Gram- was >21 days*.*

#### 3.2.3 Settling time

Storage modulus (G') and loss modulus (G") increased after mixing the solid and liquid components of Cemex; G" reached a local maximum while G' monotonically reached a plateau (Fig. 3e). The settling time (time needed for the dough to reach constant rheological properties) of Cemex was ~6 min and was not statistically impacted (*p* > 0.05) by the addition of the silica NPs (data not shown).

#### 3.2.4 Water uptake

The bone cement samples increased in weight during the first 7 days because of water uptake, and after that, the amount of water in the samples remained stable regardless of the type of NPs added (*p* > 0.05) (Fig. 3f).

#### 3.2.5 Mechanical testing

**Fig. 4:**
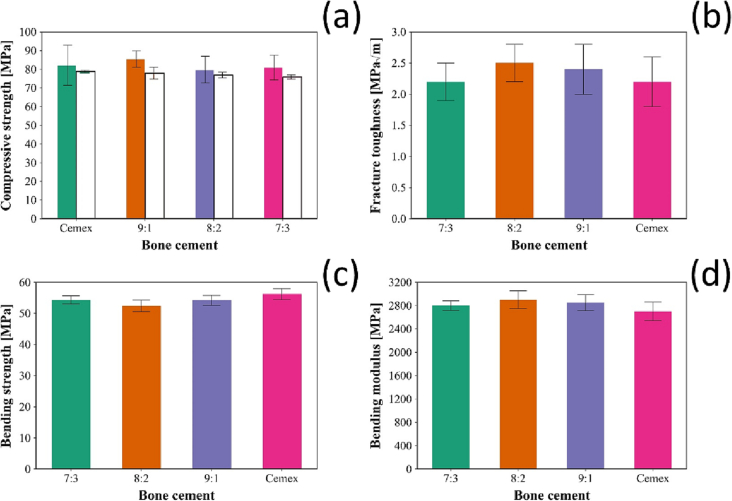
(a) Compressive strength testing for Cemex alone and with different types of NPs at zero time (coloured bars) and after 3 months (white bars) of incubation on PBS at 37 °C. (mean ± SD, *n* = 3). (b) Fracture toughness, (c) Bending strength and (d) Bending modulus for (c) Cemex () and Cemex containing different types of NPs ( 7:3,  8:2,  9:1).

The compressive strength was tested according to ISO standard 5833:2002 for Cemex and Cemex containing different types of NPs (7:3, 8:2 and 9:1) 24 h after settling and after 3 months of incubation in PBS at 37 °C (Fig. 4a). Cemex containing the different NPs developed in this study had similar compressive strength compared to Cemex (*p* > 0.05). There was no significant difference in fracture toughness (Fig. 4b) or the bending properties (Fig. 4c and d) between Cemex and Cemex with added any of the NPs prepared (*p* > 0.05). Furthermore, all types of bone cement met the criteria for in the ISO standard 5833:2002 (>70 MPa compressive strength, >50 MPa for bending strength).

#### 3.2.6 Cytotoxicity analysis

##### 3.2.6.1 MTT

**Fig. 5:**
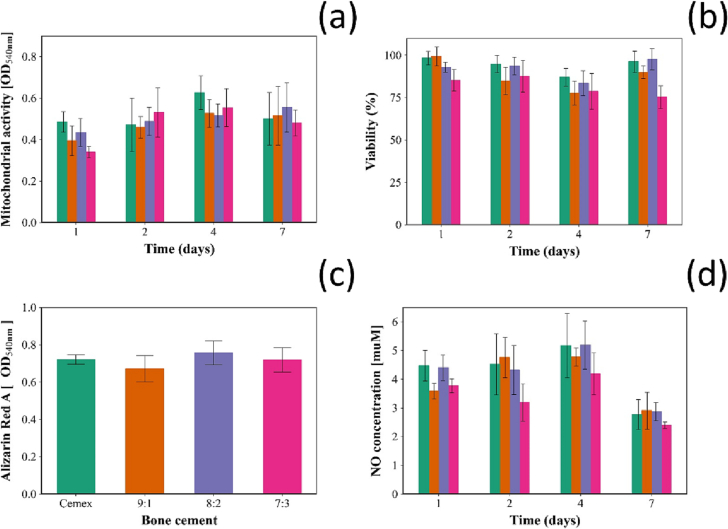
(a) Mitochondrial activity, (b) viability assessed through LDH assay, (c) Alizarin red assay after 21 days and (d) Nitrite production of Saos-2 cells exposed to different types of bone cements Cemex () and Cemex containing different types of NPs ( 7:3,  8:2,  9:1) (mean ± SD, *n* = 6).

SaoS-2 cells grown on all cements with NPs (Cemex with 9:1, 8:2 or 7:3) showed similar mitochondrial activity after incubation at all the time points tested (*p* > 0.05) compared to osteoblast grown on Cemex® (without added antimicrobial agents) (Fig. 5a).

##### 3.2.6.2 LDH

Viability of osteoblasts cells grown on Cemex® containing any of the types of NPs developed in this study was not statistically different from the same cells grown on commercial cement (*p* > 0.05) (Fig. 5b).

##### 3.2.6.3 Alizarin R

The amount of calcium deposited on the surface of the bone cements samples by growing SaoS-2 was not affected by the presence of antimicrobial releasing NPs added to the commercial formulation (*p* > 0.05) (Fig. 5c).

##### 3.2.6.4 NO production

SaoS-2 grown on Cemex containing any of the NPs tested showed the same nitrite production at different time points tested, when compared to Cemex® (Fig. 5d) (*p* < 0.05).

### 3.3 In vivo study

#### 3.3.1 Bacterial content in bone lesion

The in vivo antibacterial and regenerative effects of the implanted bone cements were studied on femoral bone lesion infected with *S. aureus*. The swab test showed a gradual reduction of viable bacteria during first 14 days after treatment of the infected bone defect (.

**Fig. 6:**
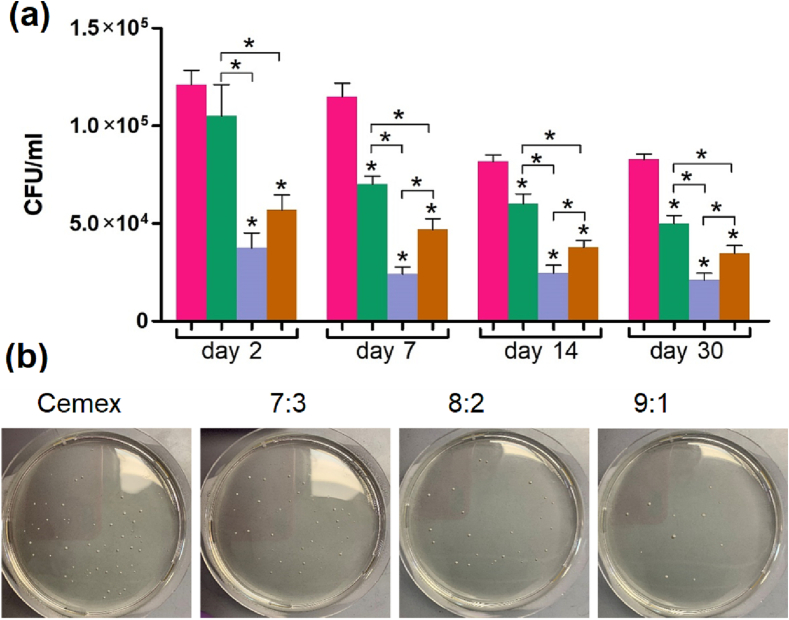
(a) Concentration of bacteria (CFU/ml) isolated from infected bone defects and representative profiles of bacterial colonies detected at day 7(cements Cemex () and Cemex containing different types of NPs ( 7:3,  8:2,  9:1). The differences in CFU values between the groups were significant (p < 0.05). (b) Representative photographs of bacterial colonies grown on inoculated agar medium in 10-cm Petri dish are also shown.

Fig. 6). There were no noticeable differences between the bacterial content at 14 and 28 days in all groups, suggesting stabilization of the bacterial infection during this period for all materials. The antibacterial effect of the bone cements was generally as follows (9:1 > 8:2 > 7:3).

#### 3.3.2 Micro-CT

**Fig. 7:**
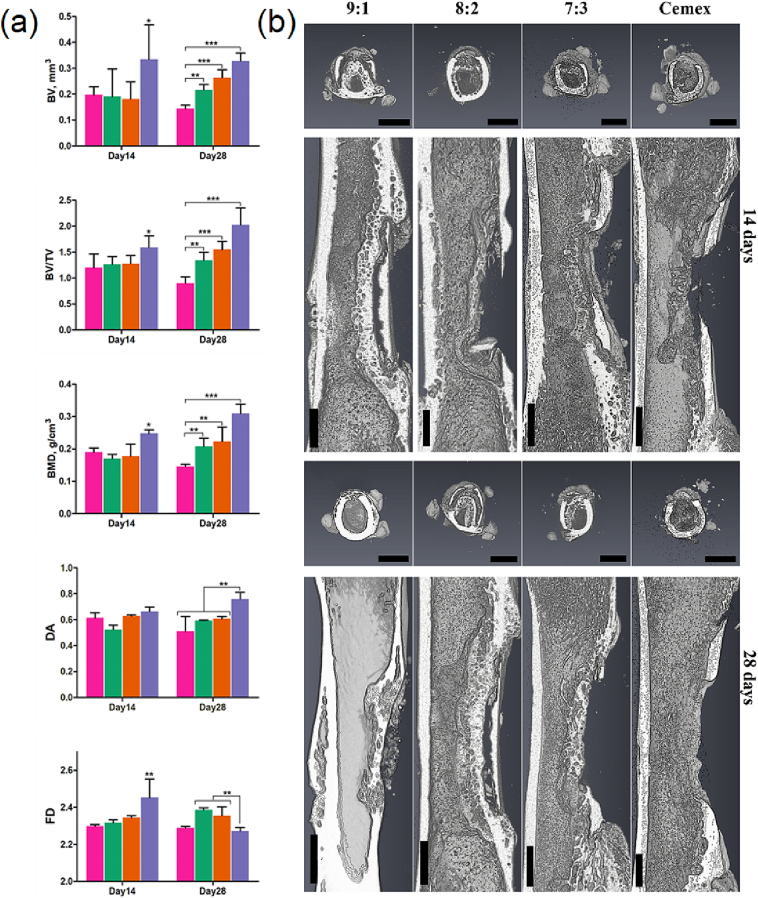
(a) Bone parameters in ROI from images analyzed using Fiji (BoneJ) software. (b) Representative micro-CT 3D images obtained using Avizo 8 software of femur bone around the infected defect (transverse and longitudinal views) after treatment with the materials (days 14 and 28) Cemex () and Cemex containing different types of NPs ( 7:3,  8:2,  9:1). Scale bar: 3 mm (transverse sections) and 1 mm (longitudinal sections).

The bone defect and surrounding bone shaft were analyzed by micro-CT at 14 and 28 days post-treatment (Fig. 7). The results confirmed that the model used is characterized by sustained fragmental lesion in the compact bone. Low-dense small voids were distributed within the compact bone around the defect presumably due to its pitting by propagating bacteria [47]. This suggests that the infection process in the bone corresponds to a diffuse type of osteomyelitis [48].

Blank Cemex group (day 14) showed negligible amount of newly formed fibrous tissues within the lesion, indicating retarded bone regeneration inhibited by the bacterial infection. In 7:3 treated group (day 14), initial fibrocartilage appeared, indicating regeneration-promoting effect of the bone cement with NPs attributed to its antibacterial activity. The other formulations exhibited even higher activity (9:1 > 8:2). In particular, the primary bone with typical woven bone-like structures with different density (calcification) began to be produced. At day 28, in the presence of bone cement containing 8:2 calcified woven bones appeared similar to those for 9:1 at day 14; whereas the bone cement with 9:1 at day 28 resulted in the beginning of bone remodeling process to form lamellar bone. Unlike 8:2 and 9:1, the other groups at day 28 showed the lack of calcification (blank Cemex) or low calcification (7:3) of the defect. Repair of the bone defect was accompanied by decrease in ‘spongy bone’ morphology in proportion to the regeneration ability of the materials so that it almost disappeared in Cemex with 9:1 group at day 28 (Fig. 7a).

Furthermore, entire bone shaft parameters within ROI were calculated from the visualized 3D bone models using established algorithms [49]. Among them, the bone volume (BV) displays the total amount of bone tissues in volume quantity; the bone volume fraction (BV/TV) displays relative overall density of these tissues; the degree of anisotropy (DA) displays bone structuration and orientation (also reflecting its strength); the fractal dimension (FD) to the contrary, displays bone irregularity and complexity.

In addition, the bone mineral density (BMD) was estimated within the lesion to reveal its calcification rate. As observed at day 28, BV, BV/TV and BMD increased in the material-treated groups with different extent. According to DA and FD, bone tissues were more structured when cement containing 9:1 was used compared to other materials (Fig. 7b). The results demonstrate favorable effect of the applied materials on the injured bone, which generally increases in the following order 7:3 < 8:2 < 9:1.

Only for blank Cemex group the above parameters showed a tendency to decrease between days 14 and 28, which was significant for BV, BV/TV and BMD (Fig. 7b). This indicates some progressing deterioration of the bone around the lesion in the absence of antibacterial attributed to sustained infection process.

#### 3.3.3 Histological assessment

**Fig. 8:**
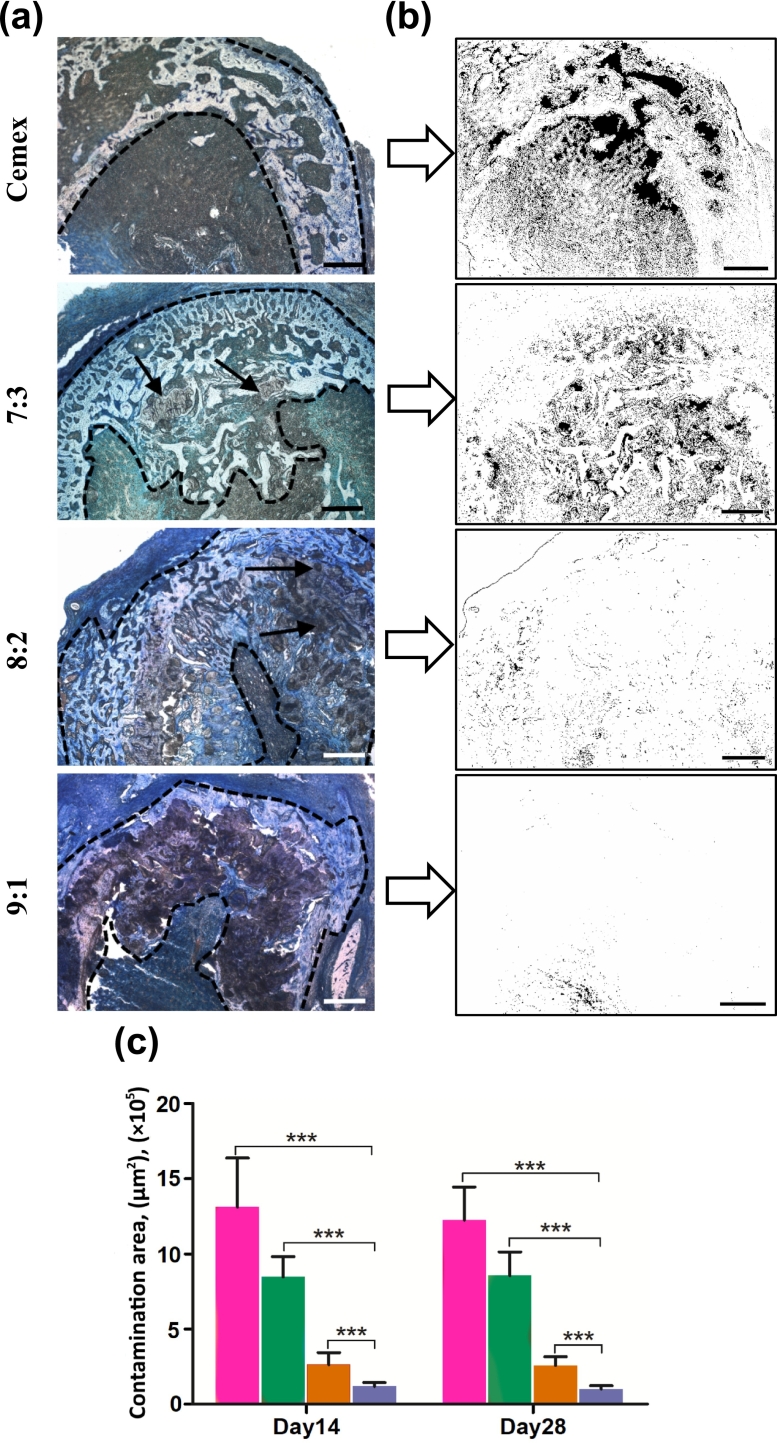
(a) Bright-field microscopy images of Giemsa stained cross-sections of infected bone defects (day 14 post-treatment), (b) Contaminated regions detected in corresponding sections according to ImageJ analysis and (c) Mean area of contaminated regions per section at days 14 and 28 (mean ± SD) Cemex () and Cemex containing different types of NPs ( 7:3,  8:2,  9:1). Scale bar represents 500 μm.

Transverse cross-sections of bone defects were subjected to Giemsa staining to visualize both bone structures and bacterial colonies (Fig. 8a). Previously, this metachromatic staining allowed us to quantify *S. aureus* infection in the skin [34]. Similarly, infected regions in the bone were identified and presented as monochromatic mask images (Fig. 8b) with mean occupied areas per cross-section (Fig. 8c).

Blank Cemex-treated bone defect featured considerable contamination throughout the section with the appearance of large and dense bacterial associates in the fibrocartilage, which is de novo formed primary compact bone (Fig. 8a and b, confined by dashed lines), further supporting profound infection process in the bone typical of osteomyelitis [47]. In 7:3 group, lower bacterial infiltration and smaller associates along with more organized fibrocartilage tissues were detected compared to blank Cemex (Fig. 8).

In comparison, 8:2 and 9:1 materials showed higher antibacterial effect, and both treated defects were composed of tissues with more intense blue color typical of non-infected bone tissues. In 8:2 group, the fibrocartilage was enlarged with pores filled by bacteria, whereas 9:1 group showed better conversion of fibrocartilage to particulate structures attributed to woven bones (Fig. 8a) Together, these data indicate that the bone cements containing NPs effectively inhibited bacterial contamination of the soft callus with their effect increased as follows 7:3 < 8:2 < 9:1 in accordance with swab test and micro-CT data.

**Fig. 9:**
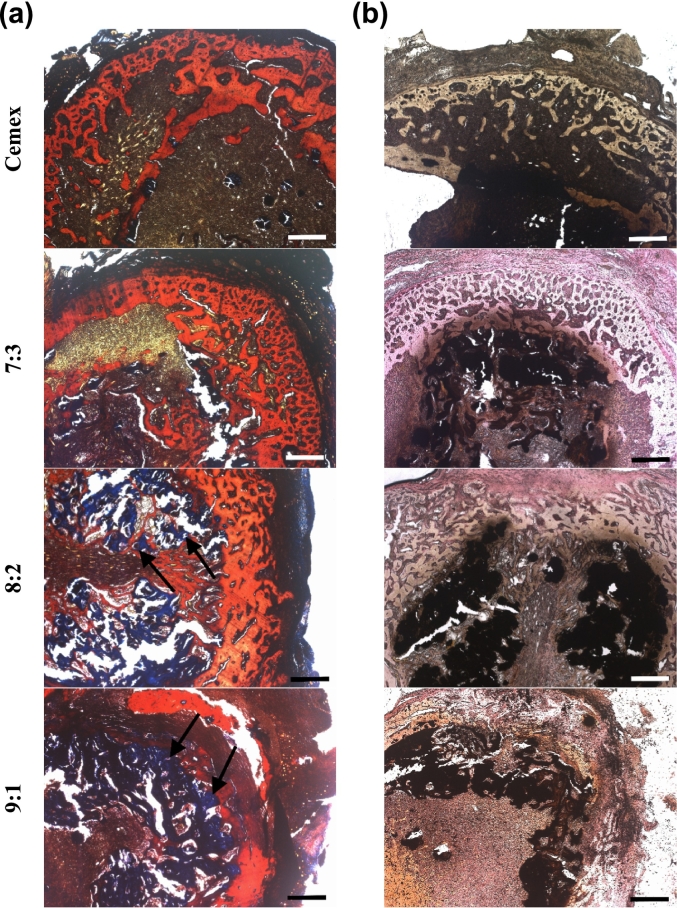
Bright-field microscopy images of (a) Picro-Mallory and (b) Von-Kossa stained cross-sections of infected bone defect (day 14 post-treatment). Scale bar represents 500 μm.

Furthermore, picro-Mallory stain was used to analyze mature collagen-containing tissues (blue) and cartilage tissues (red) in the bone callus (Fig. 9a). At day 14, both blank Cemex- and 7:3-treated bone defects showed disrupted fibrocartilage with some associated collagen depositions in the latter group. 9:1 and 8:2 groups featured higher organized fibrocartilage tissues, which were partially replaced by mature collagen fibers (9:1 > 8:2).

The collagen fibers should be associated with endochondral ossification upon the formation of primary bone [50] with typical roundish woven bone structures better appeared in 9:1 than 8:2-treated groups (Fig. 9a, arrows). The latter structures were also detected in the sections stained by Von-Kossa [51] (Fig. 9b) and micro-CT analysis (Fig. 7a).

At day 28, the histological data were generally in consistency with the micro-CT data (data not shown). In particular, according to picro-Mallory and Von-Kossa staining, blank Cemex group still showed the lack of primary bone formation in the defect, whereas in 7:3 group there were progressively formed woven bone structures, which however were non-connected between each other and separated from the cartilage.

#### 3.3.4 Chlorhexidine quantification

Blood plasma levels of antibacterials released from selected bone cements containing NPs (9:1 and 8:2) in vivo were detected on the example chlorhexidine as a predominant drug with individual structure unlike polycomponent gentamicin. MRM transitions for chlorhexidine detection were optimized using Analyst software in direct infusion mode. Based on previous data for chlorhexidine [52,53]; MRM transitions for both singly and doubly charged MS/MS ions were selected (Fig. S1). Extracted ion chromatograms for the MRM transitions of chlorhexidine standard in blank plasma and experimental samples as well as calibration curve for chlorhexidine in blank plasma are shown in Figs. S2 and S3. The lower limit of quantitation (LLOQ) for the analyte was 13.92 nM.

**Fig. 10:**
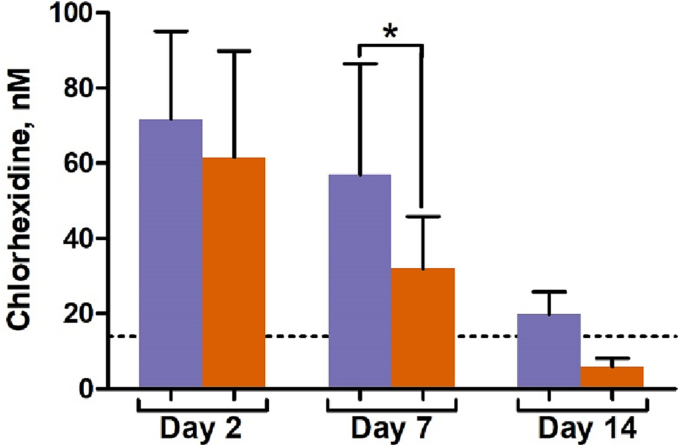
Found concentrations (mean ± SD) of chlorhexidine in blood plasma of the animals treated with Cemex containing NPs  8:2,  9:1 according to LC–MS/MS analysis. Dotted line shows LLOQ.

The results showed that both bone cements provided nanomolar systemic levels of chlorhexidine, which are far below cytotoxic concentrations; nevertheless, much higher local concentrations of the antibacterial in the bone should be expected (Fig. 10). The change in chlorhexidine levels with time suggested the ability of materials to continuously elute large amount of the drug during 7–14 days post-implantation into bone defect. At day 14, found concentrations of chlorhexidine were close to LLOQ. The LC–MS/MS data suggest that 9:1-based cement ensures significantly higher chlorhexidine level compared to that for 8:2-based counterpart (Fig. 10), and this presumably contributes to increased antibacterial and regenerative activities of the former material.

## 4 Discussion

In this work we chose silica nanoparticles because they are biocompatible and a potential inexpensive drug delivery system with high loading capacity and ease of synthesis [54]. Chlorhexidine was selected because of its non-antibiotic properties and wide used as it has many applications as a disinfectant and antiseptic for skin infections, cleaning wounds [55,56] and sterilization of surgical instruments [57,58]. Moreover, chlorhexidine is widely used in many dental applications including treatment of dental plaque, gingivitis and endodontic disease [59,60]. The use of chlorhexidine has been widely examined also in dental cements [61,62], although it has not been investigated widely in acrylic bone cements [63]; however, chlorhexidine releasing titanium has been developed to reduce infections and no adverse event were reported following in vivo validation of antimicrobial efficacy [34]. PBAE were employed as their positive charge allow entrapping both chlorhexidine and gentamicin between alginate layers (negatively charged); furthermore they are known to be biocompatible and inexpensive [6,32,34,64]. The release mechanism from drugs entrapped in LbL deposited coatings based on PBAEs is predominantly through drug diffusion through the polyelectrolyte layers despite the possible contribution of PBAE hydrolysis [32]. Once mixed in PMMA bone cements, NPs are well dispersed in the bone cement matrix [6] and act as drug reservoir with the antimicrobial drug first diffusing through the polyelectrolytes layers and then through the PMMA matrix.

TGA is a commonly used type of analysis to assess the presence of organic matter on the surface of nanoparticles, based on the observation of mass loss [65]. The thermogram for the amino functionalised silica nanoparticles (Fig. 2d) was similar to other previously presented [6,37,54]. Moreover, the calculated organic matter percentage for the amino functionalised silica nanoparticles (Fig. 2) was in agreement with other reported [6,37,54]. The consistent increase in the organic content confirmed the deposition of the layered polyelectrolytes and drugs on the surface of the amino functionalised silica nanoparticles.

Chlorhexidine and gentamicin release were studied in PBS (pH 7.3), which is the pH value in healthy joints [66]. The release from different nanocomposites (Cemex-9:1, 8:2 and 7:3) showed a reduced burst at the start of the release that continued for up to 50 days for both gentamicin and chlorhexidine (Fig. 3). The prolonged drug released is the results of the drug entrapment between multiple layers of polyelectrolyte that control the molecule availability to diffuse through the PMMA bone cement matrix [5,6,32]. The overall amount of either gentamicin or chlorhexidine increased with increasing numbers of quadruple layers confirming that the drug deposited in the inner layers remains available for release. All drug combination nanocomposites (Cemex-9:1, 8:2 and 7:3) achieved an antimicrobial inhibition up to 48 days, compared to <30 days for chlorhexidine and gentamicin nanoparticles alone [5,6,37]. This is likely the consequence of a different PBAE used in this work as the structure of the polymer is critical in controlling the release kinetic [67].

The pathogens used in this work represent the spectrum of species normally encountered in PJIs, moreover the clinical isolates were also resistant to gentamicin, and such the results clearly show how the new materials would be effective while current products would fail. Moreover, inhibition after 7–14 days (the common period of time required for conventional ALBC to stop antibiotic release) is a direct correlation to the extended drug release that the silica nanoparticles provide.

The mechanical properties of commercially available ALBC products are satisfactory, hence the addition of the silica nanocarriers developed in this work was simply required to be non-inferior to the control samples. The properties tested cover the ease of use (settling time), actual mechanical performance (fracture toughness, compression and bending strength) along with ability to support osteoblasts growth.

The changes in rheological properties during settling is critical, because implant insertion by surgeon should be delayed until the cement has a sufficient degree of viscosity, but before complete hardening of the cement [68]. The settling time can be defined as the time when the temperature of the cement reaches halfway between ambient and the peak exothermic temperatures [36]. However, using the viscoelastic parameters such as G' and G" provide a better description for the behaviour of the cement and a better measure of handling and setting characteristics [69]. The introduction of nanoparticle in the bone cement didn't change the settling time and rheological behaviour compared to the commercial cement (Fig. 3); this assures that the addition of the NPs developed in this work would not require any different handling during application. While incorporation of chlorhexidine powder in cements is commonly reported to decrease the compressive strength [70,71], the addition of the developed NPs did not compromise the mechanical properties of the nanocomposite, compared to the commercial cement (Fig. 4). The bending strength and modulus for the nanocomposite also comply with cement mechanical requirements set in ISO 5833:2002 (bending strength >50 MPa and bending modulus >1800 MPa). Water uptake of the bone cement in physiologic conditions changes the mechanical properties of the cement, because water decreases the attraction between polymer chains and increase their flexibility [72]. Therefore, studying the water uptake behaviour is important to estimate any initial changes in the physicochemical properties of the cement. The presence of NPs did not affect the water uptake compared the commercial cement, and no water was absorbed after 30 days (Fig. 3). The weight of bone cement samples stopped increasing after 4–5 days, which explains the similarity in the compressive strength after 3 months (Fig. 4).

Osteoblasts cells growth on bone cement samples was not affected by the presence of the developed silica nanocarriers due to the biological tolerability of the silica material and cytocompatibility of the drugs and both polyelectrolytes (Fig. 5). Mitochondrial activity and viability, as tested by MTT and LDH, alone do not provide a complete estimation of the potential adverse effects of the added silica nanoparticles as other biological relevant functions involved in the integration of the bone cement into the host organism are not assessed in these tests. Calcium deposition is a symbol of osteoblast's ability to promote bone formation in the period subsequent to the device implantation [73] and the developed NPs did not prevent such activity. Furthermore, nitric oxide (NO) is a free radical involved in many physiologic processes [74], such as vasodilation, inflammation thrombosis, immunity and neurotransmission; therefore, our results also demonstrate the absence of inflammatory effects in the silica nanocarriers.

We used a modified in vivo model of an infected bone defect for the purpose of a preclinical study of the effectiveness of bone cements containing the developed silica nanocarriers (Fig. 1). The previously described models of mechanical bone injury are mainly based on drilling and formation of segmental defects [75,76]. In the case of drilling, there is a risk of local burns of bone tissue, there are limitations in the diameter of the hole, usually up to 4 mm in rats, since with a larger defect against the background of a complication of a bacterial infection, the likelihood of bone fracture increases [75]. Segmental defects, including complete fractures, can be excessively traumatic for testing osteoreplacement materials, require the use of fixation devices, and are laborious to perform [76]. The model we have optimized is based on previously reported studies [40,41], however, it has a different localization of the defect on the femur, providing easier access for the formation and processing of the defect.

The model used by us, in essence, combines the advantages of drilling and segmental defects, leveling their known disadvantages. A similar defect is formed on both paws for a more relevant comparison of control and experimental samples on the same animal, which is important when using ordinary rats, characterized by variability in immune and regenerative responses, since it can significantly reduce the number of experimental animals [40]. The formed defects were effectively colonized by *S. aureus*, which is one of the main causes of infectious osteomyelitis [47,77]. The model is accompanied by the development of a rather severe form of osteomyelitis (apparently diffuse type) [48] the treatment of which, without appropriate antibiotic therapy, is difficult. The establishment of infection process in the bone was confirmed and characterized by both the direct detection of bacteria (Fig. 6, Fig. 8) and morphological analysis of bone structures impaired by bacteria (Fig. 7, Fig. 8, Fig. 9). An infected bone defect per se did not recover within a 4-week period (Fig. S4) in accordance with earlier observations that the infection process in the bone can be prolonged and irreversible [48]. Untreated infected bones featured similar morphology to that observed upon the treatment with drug-free control material (Fig. 7, Cemex). Therefore, only Cemex-treated group was used as a control to characterize drug-containing cements, which promoted repairing process to various stages depending on composition.

The selected observation days (days 14 and 28) covered the key stages preceding the complete restoration of the bone, which made it possible to compare the effect of cements on both the spread of bacteria and the formation of main bone structures. As shown previously, the swab test, though reflecting antibacterial activity of applied materials, may do not consider distribution of bacteria at injured surface and underlying tissues [34]. To analyze the distribution of bacteria in bone defect, their specific histochemical staining was carried out (Fig. 7). Relative bacterial counts were quantified by calculating the area of infection in a bone section using a procedure previously used to detect staphylococci in the skin [34]. According to both histological evaluation and swab test, the antibacterial activity of materials increases as follows 7:3 < 8:2 < 9:1.

Bone repairing activities of the materials were assessed using micro-CT and histological data. The results showed that their antibacterial activity was agreed with the efficiency of bone formation in the infected lesion. According to both analyses, the propagating infection resulted in typical spongy bone morphology of the compact bone [78] observed throughout the bone shaft around the defect. All nanocomposite formulations (Cemex-9:1, 8:2 and 7:3) inhibited the spongy bone effect in proportion to their antibacterial activity and time of treatment (14 and 28 days). In the case of the most active material, i.e., Cemex-9:1, an effective formation of calcified woven bone structures associated with endochondral ossification was observed at day 14, whereas at day 28, the remodeling process into lamellar bone began. The bone repair process in the presence of Cemex-9:1 seems to approach that in non-infected bone defect [47]. In comparison with Cemex-9:1, Cemex-8:2 and Cemex-7:3 resulted in relatively delayed endochondral ossification as revealed by less developed woven bone morphology.

Different effects of the implanted nanocomposite materials revealed suggest that these are mainly contributed by chlorhexidine component. LC–MS/MS study confirmed that Cemex-9:1 insured increased systemic and therefore local concentrations of chlorhexidine during at least 14 days of the bone treatment (Fig. 10). This also suggests that the in vivo effects seem to stronger depend on drug composition than in vitro antibacterial activity (Fig. 3) presumably due to the role of additional factors affecting local bioactivity of the implanted materials.

## 5 Conclusions

The NPs containing bone cement showed superior antimicrobial activity against different bacterial catalogue stains and gentamicin resistant clinical strains indicating that they could provide prophylaxis and treatment against PJIs after TJRs reducing the impact of such infection on patients and health care providers. The nanocomposites showed non inferior mechanical properties compared to commercial products and were cytocompatible and nontoxic to osteoblasts without adversely affecting calcium production. Moreover, efficacy against infections and safety were demonstrated in vivo using an animal model.

## Declaration of competing interest

PP is named inventor in patents related to PBAEs use in drug delivery.

## Data Availability

Data will be made available on request.

## Appendix A Supplementary data

Supplementary figures Image 37
