# Supplementary material for: Nanocomposite orthopaedic bone cement combining long-acting dual antimicrobial drugs
Source: Biomater Adv. 2023 Oct;153:None. doi: 10.1016/j.bioadv.2023.213538 (PMC10824671; doi:10.1016/j.bioadv.2023.213538)
Supplement: Supplementary file 1 — Supplementary figures [file mmc1.docx]

**Dear Editor,**

**Please find our revised manuscript and point by point referee comments replies attached alongside with the supplementary material (below).**

**Kind regards,**

**Dr Polina Prokopovich on behalf of all co-authors.**

**Supplementary material**

Nanocomposite orthopaedic bone cement combining long-acting dual antimicrobial drugs

Lirong Yang^1^, Abdulla A. Yergeshov^2^, Yazan Al-Thaher^1^, Svetlana Avdokushina^2^, Evgeny Statsenko^3^, Timur I. Abdullin^2^, Polina Prokopovich^1,*^

^1^ School of Pharmacy and Pharmaceutical Sciences, Cardiff University, Cardiff, UK

^2^ Institute of Fundamental Medicine and Biology, Kazan (Volga Region) Federal University, 18 Kremlyovskaya St., 420008 Kazan, Russia

^3^ Institute of Geology and Petroleum Technologies, 4/5 Kremlyovskaya St., 420111 Kazan, Russia

Lirong Yang and Abdulla A. Yergeshov contributed equally to the paper

* Corresponding author: Dr Polina Prokopovich

School of Pharmacy and Pharmaceutical Sciences

Cardiff University

Redwood Building, King Edward VII Avenue

Cardiff, UK

CF10 3NB

E-mail address: [prokopovichp@cf.ac.uk](mailto:prokopovichp@cf.ac.uk)

Figure S1. MS/MS spectra of (a) singly charged ion and (b) doubly charged ion of chlorhexidine.

Figure S2. Extracted ion chromatograms for MRM transitions of (a) chlorhexidine standard in blank plasma (6.96 nM) and (b) chlorhexidine in experimental samples. Retention time for chlorhexidine is 5.32 min.

Figure S3. Calibration curve for chlorhexidine in blank plasma.

|  | **(a)**   |
| --- | --- |
| **(b)**   | |

Figure S4. (a) Transverse and longitudinal micro-CT images of the infected bone (untreated control, day 14). (b) Microphotography of transsection of the infected bone stained by Giemsa (untreated control, day 14).
